# Expression Profiling of a Heterogeneous Population of ncRNAs Employing a Mixed DNA/LNA Microarray

**DOI:** 10.1155/2012/283560

**Published:** 2012-06-10

**Authors:** Konstantinia Skreka, Marek Zywicki, Michael Karbiener, Alexander Hüttenhofer, Marcel Scheideler, Mathieu Rederstorff

**Affiliations:** ^1^Section for Genomics and RNomics, Biocenter, Innsbruck Medical University, Fritz Pregl Strasse 3, 6020 Innsbruck, Austria; ^2^Institute for Genomics and Bioinformatics, Graz University of Technology, Petersgasse 14, 8010 Graz, Austria; ^3^Université de Lorraine, CNRS-UMR 7214 AREMS, 9 avenue de la Forêt de Haye, F-54506 Vandoeuvre-lès-Nancy, France

## Abstract

Mammalian transcriptomes mainly consist of non protein coding RNAs. These ncRNAs play various roles in all cells and are involved in multiple regulation pathways. More recently, ncRNAs have also been described as valuable diagnostic tools. While RNA-seq approaches progressively replace microarray-based technologies for high-throughput expression profiling, they are still not routinely used in diagnostic. Microarrays, on the other hand, are more widely used for diagnostic profiling, especially for very small ncRNA (e.g., miRNAs), employing locked nucleic acid (LNA) arrays. However, LNA microarrays are quite expensive for high-throughput studies targeting longer ncRNAs, while DNA arrays do not provide satisfying results for the analysis of small RNAs. Here, we describe a mixed DNA/LNA microarray platform, where directly labeled small and longer ncRNAs are hybridized on LNA probes or custom DNA probes, respectively, enabling sensitive and specific analysis of a complex RNA population on a unique array in one single experiment. The DNA/LNA system, requiring relatively low amounts of total RNA, which complies with diagnostic references, was successfully applied to the analysis of differential ncRNA expression in mouse embryonic stem cells and adult brain cells.

## 1. Introduction

The high-resolution analysis of 1% of the human genome by the ENCODE project has shown that up to 90% of the genome is being transcribed while only about 1.5% of these transcripts correspond to protein coding exons [1]. Therefore, it was suggested that the majority of the transcripts might serve as a source for regulatory non coding RNAs (ncRNAs) [2, 3], with the predicted number of ncRNAs present in the human genome reaching up to 0.5 million transcripts [4]. However, most of these transcripts still remain of unknown function, and their functionality is even debated [4].

These novel exciting aspects of the cellular transcriptome content thus require novel methods for profiling ncRNAs expression in a high-throughput manner. Lately, the most widely used expression profiling technique has become high-throughput sequencing or RNA-seq [5, 6], with numerous advantages. RNA-seq provides full genome coverage and allows detection of single nucleotide polymorphisms as well as RNA editing events, independently of hybridization artifacts. However, RNA-seq drawbacks and artifacts are not completely absent, generally linked to reverse transcription or library generation protocols [6, 7]. In addition, analysis of sequencing datasets is still rather time consuming and requires a strong bioinformatic expertise, which does not make it suitable for rapid diagnostic or clinical profiling so far. An alternative high-throughput approach is based on microarrays. Recently, novel microarray technologies have evolved to efficiently profile miRNA expression [8, 9] or detect single nucleotide polymorphisms [10] by employing locked nucleic acid (LNA) arrays. LNAs are synthetic RNA analogs characterized by increased thermostability of nucleic acid duplexes, allowing increased hybridization temperatures and thus improved mismatch discrimination [11]. With the recent interest in ncRNAs as biomarkers [12–14], ncRNA microarrays might represent a suitable tool to profile ncRNA expression for diagnostic purposes. However, an LNA platform would not be generally financially affordable for these applications. Here, we describe a mixed DNA/LNA microarray platform that allows the hybridization of directly and simultaneously labeled small and longer ncRNAs onto microarrays consisting of both LNA-modified and custom-designed DNA capture probes, respectively. This method enables a sensitive and specific analysis of a complex and heterogeneous RNA population on a unique array in one experiment, complying with nowadays most criteria in biomedical diagnostics in terms of cost and sample requirements.

## 2. Materials and Methods

### 2.1. Probes

The miRCURY LNA miRNA array ready-to-spot probe set (reference 208010) was purchased from Exiqon (Denmark) as an LNA capture probe set for short ncRNAs detection. This set comprises 2,056 capture probes designed to have a uniform *T*_*m*_ of 72°C and covers all miRNAs of miRBase (version 9.2).

The DNA probes were purchased from Microsynth (Switzerland). They were 5′-C6 amino-modified, designed so as to comply with a 72°C *T*_*m*_, desalted and diluted in 3xSSC, 1.5 M Betaine buffer to a final concentration of 20 *μ*M.

### 2.2. ncRNA Chip

The LNA-based capture probe set for short ncRNAs as well as the self-designed DNA-based capture probe set for long ncRNAs was spotted on HiSens epoxy-coated glass slides (Nexterion) using the MicroGrid II Microarray Spotter (Zinsser Analytic). Every probe (antisense, mismatch, deletion, and sense) was spotted twice on the slide in four replicates (local separation) to ensure quality assurance and reliability.

### 2.3. Hybridization Station

Hybridizations have been performed using the Tecan HS400 hybridization station according to the Exiqon protocol for hybridization with the miRNA LNA platform. Hybridizations were performed at 56°C or 64°C.

### 2.4. Microarray Scanner

The ncRNA chip was scanned using the Axon instruments GenePix 4000B.

### 2.5. RNA Labeling

Total mouse brain RNA was extracted from C57/Bl6 mice (4–8 weeks old) and total mouse embryonic stem cell RNA from E14 stem cells with TriReagent (Sigma-Aldrich) following the manufacturer's protocol. RNA was quantified employing a nanodrop spectrophotometer (Fischer Scientific). Total mouse brain RNA (0.25 *μ*g–5 *μ*g) and total mouse embryonic stem cell RNA (2 *μ*g) were directly labeled employing the NCode Rapid miRNA Labeling System (Invitrogen), following the manufacturer's protocol with the following modifications: (i) prior to poly-A tailing, RNA was denatured at 90°C for 3 min, centrifuged, and cooled on ice for 2 min and (ii) the reaction buffer provided with the kit was replaced by a custom reaction buffer containing 50 mM Tris-HCl (pH 8.0), 250 mM NaCl, and 10 mM MgCl_2_. For differential expression, 2 biological replicates of total mouse brain RNA and total mouse embryonic stem cell RNA were used.

### 2.6. Probe Design

Probes were designed employing OligoWiz. Post-processing steps, including verification of probes specificity or processing events coverage, were added. For a full description, see [15].

### 2.7. Northern Blot

Northern blots were performed as described previously [16]. Following oligonucleotides (Sigma-Aldrich) were used: SNORA71 5′-TATCAATGACCAGGGCACCCGCAGCCC-3′, SNORD55 5′-GTCGGGAGTGTGCAGCATACCCAGGTG-3′ and 5′-GCAATTCACATTAATTCTCGCAGCTAGC-3′.

### 2.8. Real-Time PCR

Total RNA was isolated from mouse ES cells and mouse brain of C57/Bl6 mice, 4–6 weeks old, with TRI Reagent (Sigma-Aldrich, Vienna, Austria) according to the manufacturer's protocol. Five hundred nanograms of total RNA were poly-A tailed and reverse transcribed to cDNA using the microRNA 1st strand synthesis kit (Agilent Technologies, Böblingen, Germany) following the manufacturer's protocol. 

The cDNA was used as template for the real-time PCR. The universal reverse primer provided with the kit was used together with the following forward primers: mmu-miR-125-5p 5′-TCCCTGAGACCCTAACTTGTGA-3′, mmu-miR-293 5′-AGTGCCGCAGAGTTTG-TAGTGT-3′, SNORD113 5′-GGGTGCTGTATGAGTCGTGTATTATGA-3′, 7SK 5′-CCATTGTAGGAGAACGTAGGGTAG-3′, SNOZ39 5′-TGATGAAGCAAATCAGTATGAATAAAATG-3′, SNORA18 5′-TGACTCACAGGACTGACTGTTAGGCCTG-3′, SNORD55 5′-CACCTGGGTATGCTGCACACTCC-3′, SNORA71 5′-CTGCCGGTGCCCTGGTCATTG-3′, U6 5′-CTCGCTTCGGCAGCACA-3′.

Primers were purchased from Sigma-Aldrich. Real-time PCR was performed using Power SYBR Green PCR Master Mix (Applied Biosystems, Darmstadt, Germany). Reactions were performed for 40 cycles with annealing step temperature set at 60°C. All results from three technical replicates were normalized to U6 and expressed as ΔCt values. Relative expression ratios were calculated by the ΔΔCt method [17]. Five independent biological samples from either mouse brain or mouse ES cells contributed to the data set. Data are presented as mean ± standard error of the mean (SEM). Student *t*-test was applied to compare between two groups. Differences were considered significant when *P* < 0.05.

## 3. Results and Discussion

### 3.1. Development of a Mixed DNA/LNA Microarray

We investigated whether a combined DNA and LNA platform, dedicated to the expression analysis of long ncRNAs as well as small ncRNAs, respectively, could be used for the expression profiling of a heterogeneous population of ncRNAs. To that end, we generated a custom microarray spotted with (i) DNA capture probes for tRNAs, 7SK RNA as well as C/D and H/ACA box snoRNAs (Supplementary Table  1) and (ii) the commercially available miRCURY LNA miRNA ready-to-spot probe set from Exiqon. To generate a mixed DNA/LNA microarray, all probes spotted had to exhibit the same melting temperature. We opted for a fixed hybridization temperature to avoid elevated background due to unspecific hybridization, as observed when using temperature gradients [18] (see the Supplementary Material available online at doi:10.1155/2012/283560). As the LNA capture probe set melting temperature corresponds to 72°C for an optimal hybridization temperature of 64°C, DNA capture probes were designed to comply with this criterion, independently of their sizes. DNA capture probes were designed to hybridize to conserved regions of ncRNAs, spanning regions of 30 to 60 nt (see Section 2). 7SK RNA and tRNAs were chosen to test hybridization capabilities for highly structured ncRNAs and snoRNAs to check for the system sensitivity. Two or more DNA capture probes were designed per ncRNA if the length of the target was sufficient (Supplementary Table  1). Additionally, in order to test the specificity of the system, probes bearing one or two nucleotides mismatches were designed in addition to the perfect matching antisense probes. Finally, for more structured ncRNAs, probes with one or two nucleotides deletions were designed (Supplementary Table  1). Sense probes for each ncRNA and random DNA probes were included as negative controls.

### 3.2. Direct RNA Labeling

**Figure 1:**
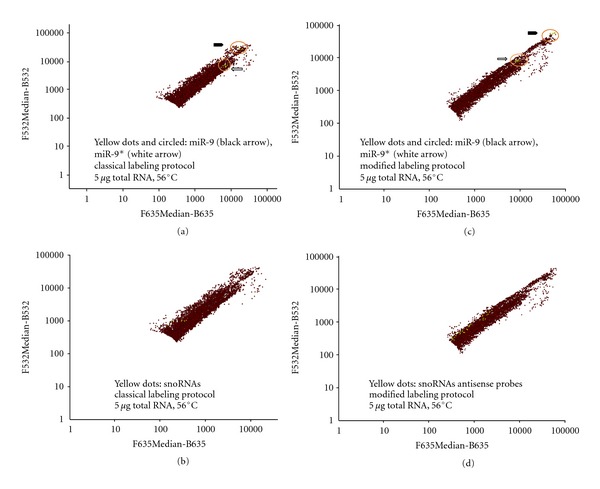
Self-self hybridization at 56°C of 5 *μ*g (per dye) of labeled total mouse brain RNA. Diagrams of average intensity values show filtered results in logarithmic scale. The *y* axis represents values of AlexaFluor3 dye measurement at 532 nm, and the *x* axis represents values of AlexaFluor5 dye measurement at 635 nm. Red spots represent all signals from LNA and DNA probes spotted on the microarray slide. (a, c) Detection of MiR-9 (yellow spots, black arrow) and miR-9* (yellow spots, white arrow) with LNA probes. (b, d) Detection of snoRNAs (yellow spots) with antisense DNA probes (including mismatch probes in (d)). Sense DNA probes signals were below detection levels and thus filtered out. (a, b) RNA labeling using the protocol described by manufacturer. (c, d) Modified RNA labeling (see Section 2).

**Figure 2:**
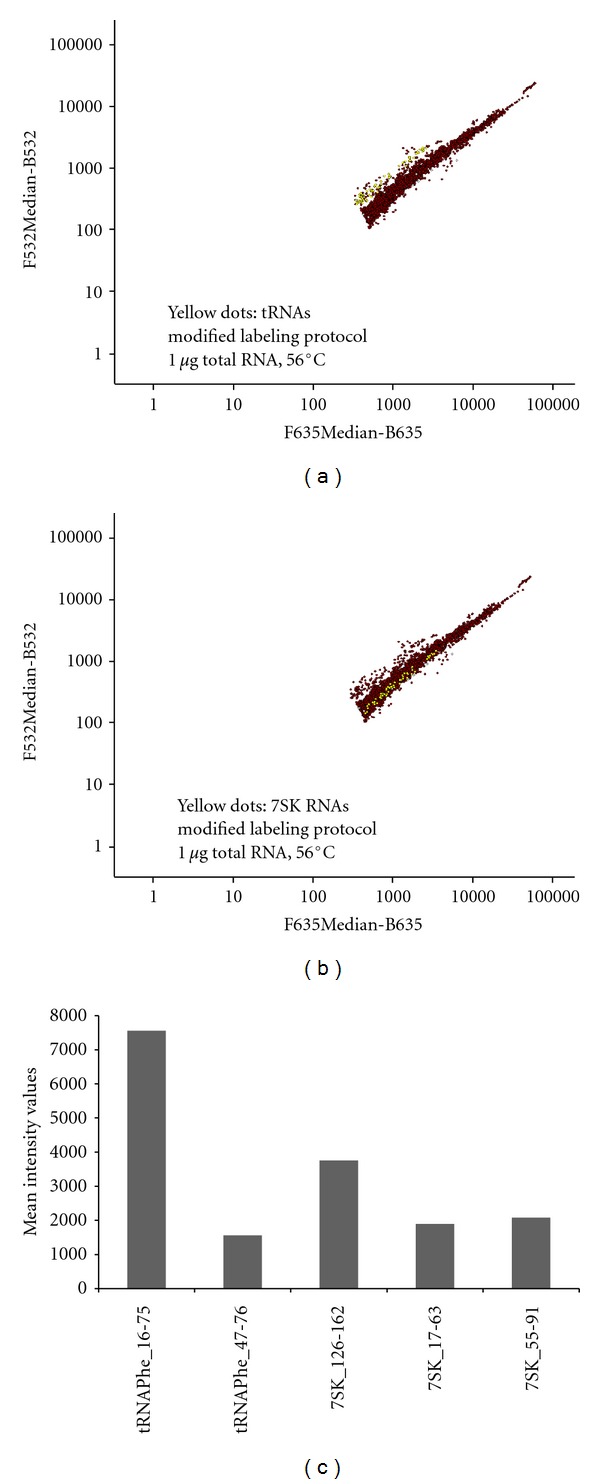
Self-self hybridization at 56°C of 1 *μ*g (per dye) of labeled total mouse brain RNA. Diagrams of average intensity values show filtered results in logarithmic scale. The *y* axis represents values of AlexaFluor 3 dye measurement at 532 nm, and the *x* axis represents values of AlexaFluor5 dye measurement at 635 nm. Red spots represent all signals from LNA and DNA probes spotted on the microarray slide. Detection of (a) tRNAs (yellow spots) and (b) 7SK RNA (yellow spots) with antisense DNA probes (including mismatch and deletion probes). Sense DNA probes were below detection levels and thus filtered out. (c) Diagram showing mean intensity values (*y* axis) of DNA probes for detection of highly structured ncRNAs.

The choice of the RNA labeling method had to be addressed. Indeed, for microarray assays, small RNAs are generally directly labeled while longer RNAs are generally reverse transcribed into cDNA and labeled through incorporation of aminoallyl-modified nucleotides. We employed a commercially available dual fluorescent dye RNA labeling kit based on poly-A tailing and ligation of fluorophore-bearing dendrimers (see Section 2). We used 5 *μ*g total mouse brain RNA for our initial proof of concept experiments. To exclude dye bias effects [19], AlexaFluor3 and AlexaFluor5 labeled total mouse brain RNA replicas were self-self hybridized on the custom DNA/LNA chip. Analysis of the results showed that neural miRNAs such as miR-9 and miR-9* were well detected (Figure 1(a)) in contrast to snoRNAs which were almost undetectable (Figure 1(b)). 7SK RNA was only marginally detectable (Supplementary Figure  1(a)) while tRNAs were almost not detectable at all. As insufficient detection of these longer ncRNAs could be linked to secondary structure-related inefficient polyadenylation and labeling, we introduced a denaturation step prior to poly-A tailing. Additionally, as Mn^2+^ cations were reported to stimulate unspecific activity of poly-A polymerase activity *in vitro *[20], we tested a Mg^2+^ custom poly-A tailing buffer (see Section 2), which increased efficiency of labeling, most likely by stimulating polyadenylation. The improved labeling protocol enabled enhanced snoRNAs, tRNAs, and 7SK RNA detection (cf. Figures 1(d) and 1(b), cf. Supplementary Figures  1(b) and 1(a), Figures 2(a) and 2(b) and Supplementary Figure  1(c)) without altering detection of miRNAs (cf. Figures 1(a) and 1(c)). Finally, posttranscriptional RNA modifications [21], such as pseudouridylation or 2′O-methylation [22, 23], might interfere with labeling of ncRNAs; we did not investigate, however, the extent of this parameter.

### 3.3. Optimization of RNA Quantity and Hybridization Temperature

**Figure 3:**
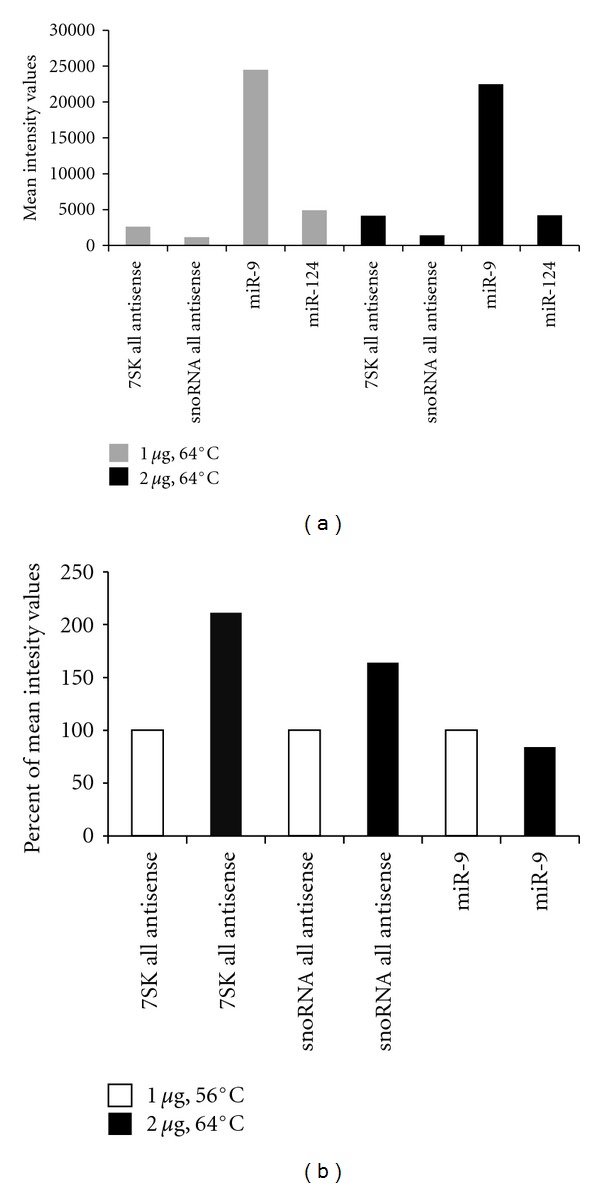
(a) Mean intensity values of all antisense DNA probes detecting snoRNAs and 7SK RNA and LNA probes detecting mir-9 and miR-124 when 1 *μ*g (grey bars) or 2 *μ*g (black bars) of labeled total mouse brain RNA is used with a hybridization temperature of 64°C. (b) Percentage of mean intensity values for all antisense DNA probes detecting snoRNAs and 7SK RNA and LNA probes detecting mir-9. White bars: hybridization at 56°C with 1*μ*g of labeled total mouse brain RNA; black bars: hybridization at 64°C with 2 *μ*g of labeled total mouse brain RNA.

Together with RNA labeling it was also necessary to optimize RNA quantity used for labeling. As few as 30 ng total RNA are generally sufficient for hybridization on LNA microarrays, while DNA microarrays require at least 10–25 *μ*g of total RNA as starting material for cDNA labeling through reverse transcription. Amounts of total RNA ranging from 0.25 to 1 *μ*g per labeling reaction were used, with a first hybridization temperature of 56°C. Under these conditions, labeling of total RNA quantities below 1 *μ*g provided insufficient results (data not shown) while labeling of 1 *μ*g of RNA resulted in satisfying results (Figures 2(a) and 2(b)). Next, the hybridization temperature was raised to 64°C to better comply with the LNA platform. In order not to compromise the sensitivity at this temperature, the quantity of labeled total RNA was raised from 1 *μ*g to 2 *μ*g. In these conditions, we observed similar results regarding miRNA LNA probes, but improved detection in the case of the 7SK RNA and snoRNAs (Figures 3(a) and 3(b)). We generally observed improved detection at 64°C for the DNA probes compared to the 56°C condition, while LNA probes remained unaffected (Figure 3(b)). However, as for tissue profiling, large quantities of total RNA might not be available, we opted for 2 *μ*g of total RNA per labeling reaction, with self-self hybridizations performed at 64°C, which appeared as the best compromise. For diagnostic purposes with lower amounts of material, further optimization of the protocol might be needed.

### 3.4. Sensitivity and Specificity of the Mixed DNA/LNA Microarray

We next tested the influence of ncRNA structure on the sensitivity and specificity of hybridization on the DNA/LNA combined platform. At 56°C, expression of highly structured RNAs, such as tRNAs and 7SK RNA, could be detected (Figures 2(a) and 2(b)). For example, tRNA^Phe^ was detected with a mean intensity of 7555 with the 60 nt long capture probe tRNAPhe_16-75, but with a reduced mean intensity of 1564 with the 30 nt long probe tRNAPhe_47-76 (Figure 2(c)). On the other hand, detection of 7SK RNA was almost 2-fold higher with the 36 nt long capture probe 7SK_126-162 compared to the probes 7SK_17–63 and 7SK_55–91 of 46 and 36 nucleotides in length, respectively, (Figure 2(c)). Thus, detection of highly structured ncRNAs appears rather independent of the capture probe's length, and employing multiple probes complementary to one particular RNA therefore increases sensitivity of detection.

**Figure 4:**
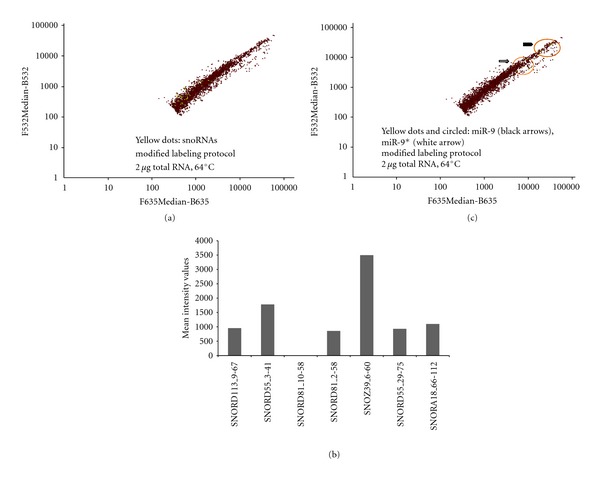
Self-self hybridization at 64°C of 2 *μ*g (per dye) of labeled total mouse brain RNA. Diagrams of average intensity values show filtered results in logarithmic scale. The *y* axis represents values of AlexaFluor3 dye measurement at 532 nm, and the *x* axis represents values of AlexaFluor5 dye measurement at 635 nm. Red spots represent all signals from LNA and DNA probes spotted on the microarray slide. (a) Detection of snoRNAs (yellow spots) with antisense DNA probes. (b) Detection of miR-9 (yellow spots, black arrows) and miR-9* (yellow spots, white arrow). Sense DNA probes were below detection levels and filtered out. (c) Diagram showing mean intensity values (*y* axis) of all antisense DNA probes detecting snoRNAs.

At 64°C, the results showed that antisense snoRNA capture probes detected efficiently snoRNAs (Figure 4(a)) with similar intensities compared to the condition where higher amounts of labeled total RNA and lower hybridization temperatures were employed (Figure 1(d)). Moreover, almost all antisense snoRNA capture probes detected their specific snoRNA but with different intensities (Figure 4(b)), while the detection levels of miR-9 and miR-9* remained identical (Figure 4(c)).

**Figure 5:**
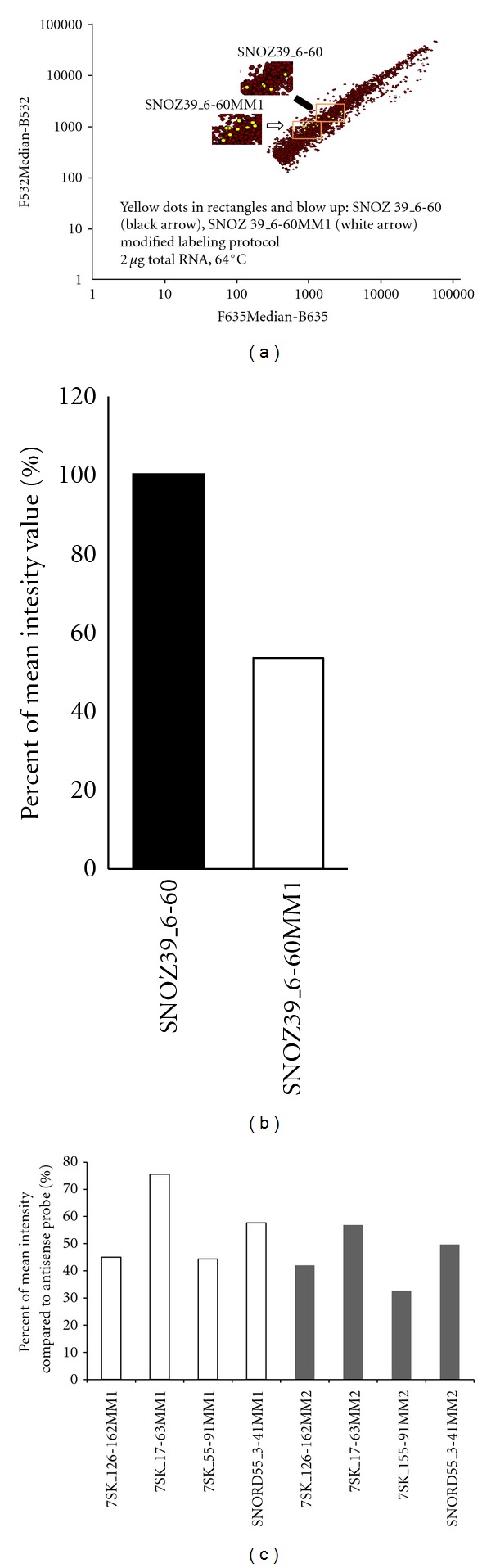
Self-self hybridization at 64°C of 2 *μ*g (per dye) of labeled total mouse brain RNA. Diagrams of average intensity values show filtered results in logarithmic scale. The *y* axis represents values of AlexaFluor3 dye measurement at 532 nm, and the *x* axis represents values of AlexaFluor5 dye measurement at 635 nm. Red spots represent all signals from LNA and DNA probes spotted on the microarray slide. (a) Detection of snoZ39 (yellow spots) with antisense DNA probes (black arrow, blow up) and one nucleotide mismatch DNA probe (white arrow, blow up). (b) Diagram showing on the *y* axis the percentage of mean detection value of the antisense probe SNOZ39−6–60MM1 and the one nucleotide mismatch probe SNOZ39−6–60MM1. (c) Diagram showing the mean intensity values (*y* axis) of the antisense probes of one (MM1) and two (MM2) nucleotide mismatch probes for 7SK RNA and SNORD55.

Discrimination at the nucleotide scale is possible with LNA capture probes. Therefore, we wanted to test how specific the detection with DNA capture probes can be with the DNA/LNA platform. The specificity was therefore checked employing also probes with mismatches at one (MM1) or two (MM2) positions. At 64°C, the snoRNA SNOZ39 was only detected by the antisense and one nucleotide mismatch capture probes, while the signal with the two nucleotides mismatch probe SNOZ39_6-60MM2 was falling below threshold (Figure 5(a)), indicating that discrimination was already possible with two nucleotides mismatches. However, the comparison of the mean intensity values between the perfect matching and one nucleotide mismatch probes showed a reduction of 40% for the MM1 probe for detection of SNOZ39 (Figure 5(b)). In some cases though, a signal was still detectable with MM2 probes (7SK RNA or snoRNA SNORD55, Figure 5(c)), but with reduced intensities compared to the perfect matching capture probes. For instance, 7SK_17–63MM2 and 7SK_55–91MM2 showed a further 20% reduction in intensity levels compared to the MM1 capture probes (Figure 5(c)).

### 3.5. DNA/LNA Platform Accuracy for Expression Profiling

**Figure 6:**
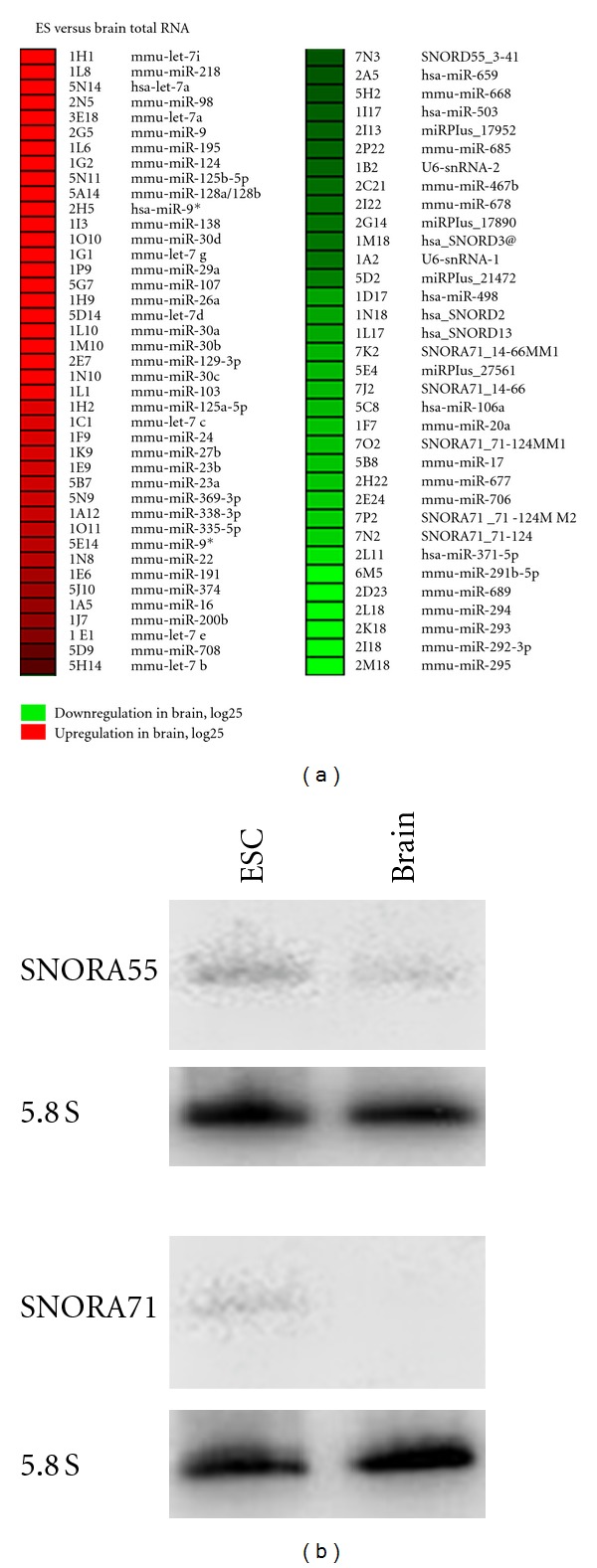
(a) Heat map showing differential expression between brain and mouse embryonic stem cells of ncRNAs spotted on the DNA-LNA microarray. Up- and downregulation of ncRNAs in brain are indicated with red or green color, respectively. Only differential expression of at least two folds is indicated. (b) Northern blot showing expression of SNORD55 and SNORA71 in mouse embryonic stem cells and mouse brain. Ten micrograms of total RNA were used, and 5.8S rRNA was used as a loading control.

We next applied our DNA/LNA platform to expression profiling, employing dye swap experiments with 2 *μ*g of total mouse brain RNA and 2 *μ*g of total mouse embryonic stem cells RNA. Hybridizations were performed at 64°C. As expected, differences in ncRNA expression between mouse adult brain RNA and mouse embryonic stem cell RNA could be detected (Figure 6). For example, stem cell specific miRNAs of the miR-290 family (miR-291–295, [24]) were detected to be about 25-fold overexpressed in mouse embryonic stem cells, while brain-specific miR-124 and miR-9 were about 14-fold overexpressed in mouse brain (Supplementary Table  2). Additionally, the brain-enriched miR-125b-5p was also about 13.5-fold overexpressed. Also let-7 was overexpressed in brain compared to ES cells, which was expected, since mature let-7 is expressed upon stem cell differentiation into neural cells (data not shown) [25, 26]. Regarding DNA probes, overexpression of SNORD55 by about 2-fold and SNORA71 by about 5-fold in mouse embryonic stem cells (Figure 6 and Supplementary Table  2) could be observed. This differential expression was confirmed by northern blots (Figure 6(b)) where SNORD55 and SNORA71 appear overexpressed in embryonic stem cells in comparison to mouse brain.

**Figure 7:**
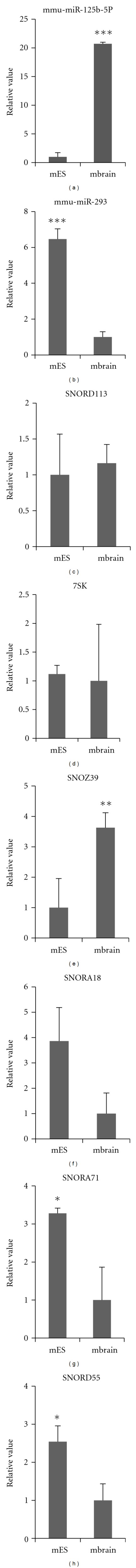
Real-time PCR verification of differential expression of selected ncRNAs captured by DNA and LNA probes on the DNA/LNA microarray platform. Results are represented as relative expression levels between mouse ES cells and mouse brain. Data are shown as mean ± SEM; *n* = 5; *P < 0.05; **P < 0.01, ***P < 0.005  significantly different from mES cells by Student *t*-test.

We employed real-time PCR to verify the differential expression observed and validate the DNA/LNA platform. We analyzed both microRNAs and ncRNAs captured by DNA probes for verification. As expected from the microarray data, we verified that miR-125b-5p and miR-293 were significantly overexpressed in mouse brain and mouse ES cells, by 20.7-fold and 6.5-fold, respectively (Figure 7). The snoRNAs SNORA71 and SNORD55 were verified to be significantly overexpressed in mouse ES cells in comparison to mouse brain as well, by 3.3- and 2.5-fold, respectively (Figure 7). According to the DNA/LNA microarray platform data, we did not observe any differential expression for SNORD113 or 7SK RNA between mouse ES cells and mouse brain (Figure 7). Finally, while they did not appear to be differentially expressed in the microarray data, SNORA18 and SNOZ39 seemed to be overexpressed in mouse ES cells or in mouse brain, respectively, according to real-time PCR results (Figure 7). While this differential expression was not significant for SNORA18, it reached 3.6-fold for SNOZ39, highlighting the necessity to validate microarray data, as well as deep-sequencing data, by additional means like northern blotting or real-time PCR. Nevertheless, our observation of differential expression of canonical snoRNAs constitutes an exciting aspect, especially regarding their recently described noncanonical functions as miRNA precursors or regulators of alternative splicing [27–31].

## 4. Conclusion

NcRNAs are now widely considered as excellent disease biomarkers [32]. For instance, miRNAs [14, 33], snoRNAs [12] or long interspersed noncoding RNAs (lincRNAs) [34] can be employed to determine the origin of various cancers. Noncoding RNAs have also been shown to be involved in chromatin regulation [35] or in neurological diseases [36, 37]. For diagnostic purposes, microarrays still appear as a less expensive method compared to high-throughput sequencing and, additionally, microarray analysis requires significantly less time. However, a microarray platform enabling simultaneous analysis of both small and long molecules for ncRNA-based diagnostic or expression profiling was lacking. Here we developed a microarray platform where both small and long ncRNAs can be profiled on the same chip. The size limitation of small RNAs prompted us to employ the already available LNA platform for miRNAs, to combine it with custom DNA capture probes for longer ncRNAs and to allow detection of all ncRNAs with a universal direct labeling procedure. Hence, long, structured ncRNAs and miRNAs could be detected with the DNA/LNA platform, and this detection was independent of the capture probe length but rather depending on secondary structure. We observed that capture probes were more efficient when designed to hybridize to less structured regions. In case of highly structured tRNAs, capture probes spanning almost the entire molecule appeared to be the most efficient. The mixed microarray is sensitive and specific and requires relatively low amounts of directly labeled total RNA. Problems due to ncRNA structure can be solved if probes are designed to span low structured regions and if a denaturation step is introduced prior to RNA direct labeling. Hence, this platform might become a very attractive tool for combined expression profiling of small and long ncRNAs as well as in biomedical diagnostic.

## Supplementary Material

Supplementary Figure 1: Self-self hybridization at 56°C of 5 *μ*g (per dye) of labeled total mouse brain RNA. Diagrams of average intensity values show filtered results in logarithmic scale. The y axis represents values of AlexaFluor3 dye measurement at 532 nm and the x axis represents values of AlexaFluor5 dye measurement at 635 nm. Red spots represent all signals from LNA and DNA probes spotted on the microarray slide. Detection of 7SK RNA from antisense probes (a) without any modifications to the RNA labeling protocol, or (b) with the modified labeling protocol (see results). (c) Detection of tRNAs with the modified labeling protocol (see results).
Supplementary Table 1: Sequences of the ncRNA corresponding DNA probes spotted on the microarray. Nucleotide mismatches are shown in bold and positions with deletions are underlined. Hybridization position of the probe on the ncRNA is indicated with numbers at the end of the probes name. Abbreviations: MM1: one nucleotide mismatch; MM2: two nucleotides mismatches; D1: one nucleotide deletion; D2: two nucleotides deletions; RC: sense probe.
Supplementary Table 2: Filtered results corresponding to Figure 6. Fold changes are indicated as log2 values. Only ncRNAs differentially expressed above 2 folds are mentioned.
