# Supplementary material for: Expression Profiling of a Heterogeneous Population of ncRNAs Employing a Mixed DNA/LNA Microarray
Source: J Nucleic Acids. 2012 Jun 10;2012:283560. doi: 10.1155/2012/283560 (PMC3384982; doi:10.1155/2012/283560)
Supplement: Supplementary file 1 — Supplementary Figure 1: Self-self hybridization at 56°C of 5 μg (per dye) of labeled total mouse brain RNA. Diagrams of average intensity values show filtered results in logarithmic scale. The y axis represents values of AlexaFluor3 dye measurement at 532 nm and the x axis represents values of AlexaFluor5 dye measurement at 635 nm. Red spots represent all signals from LNA and DNA probes spotted on the microarray slide. Detection of 7SK RNA from antisense probes (a) without any modifications to the RNA labeling protocol, or (b) with the modified labeling protocol (see results). (c) Detection of tRNAs with the modified labeling protocol (see results). Supplementary Table 1: Sequences of the ncRNA corresponding DNA probes spotted on the microarray. Nucleotide mismatches are shown in bold and positions with deletions are underlined. Hybridization position of the probe on the ncRNA is indicated with numbers at the end of the probes name. Abbreviations: MM1: one nucleotide mismatch; MM2: two nucleotides mismatches; D1: one nucleotide deletion; D2: two nucleotides deletions; RC: sense probe. Supplementary Table 2: Filtered results corresponding to Figure 6. Fold changes are indicated as log2 values. Only ncRNAs differentially expressed above 2 folds are mentioned. [file 283560.f1.pdf]

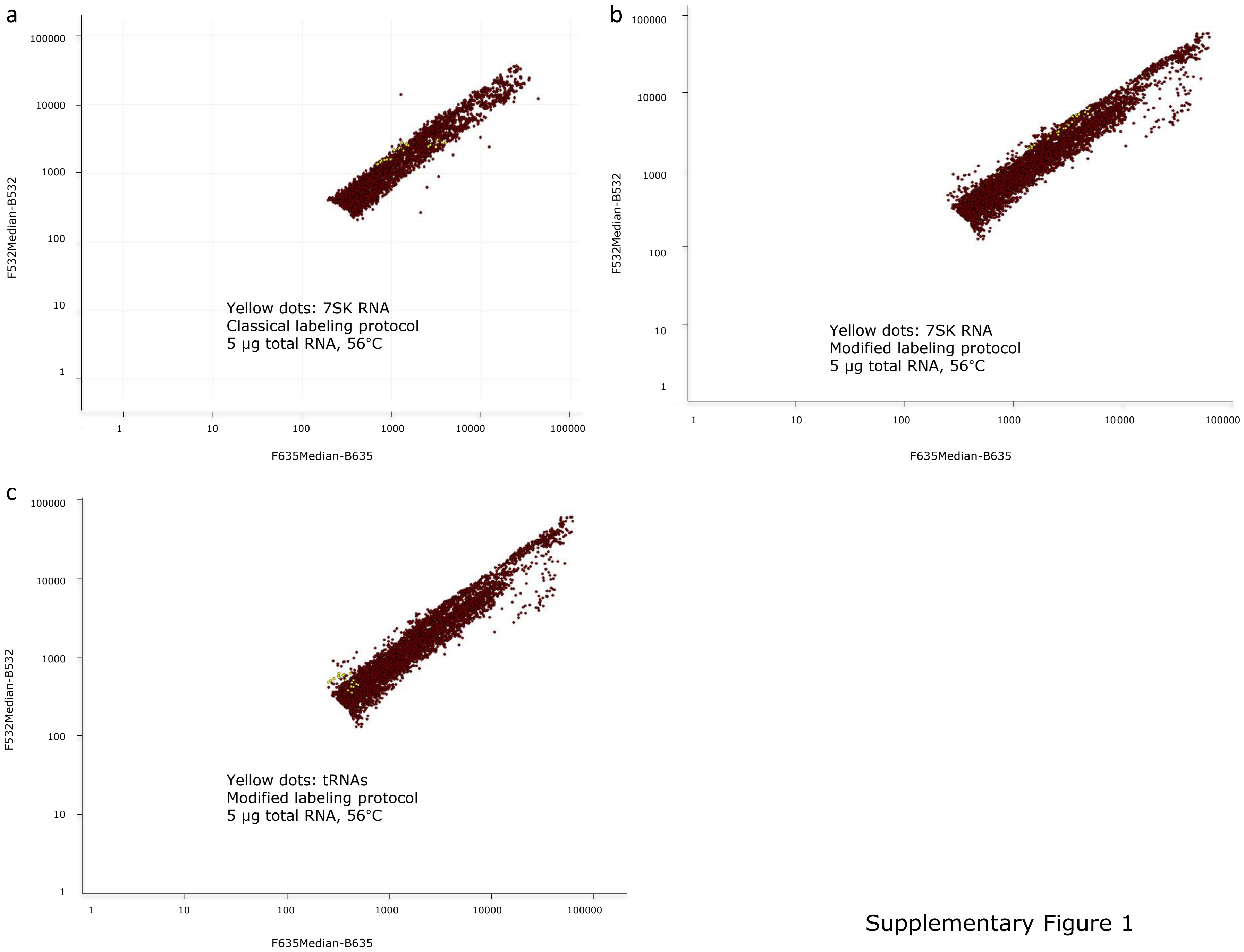


Supplementary Figure 1

| Oligo name | Sequence (5' → 3') |
| --- | --- |
| 7SK_126-162 | TCGGGGAAGGTCGTCCTCTTCGACCGAGCGCGCAGCT |
| 7SK_126-162D1 | TCGGGGAAGGTCGTCCTCTCGACCGAGCGCGCAGCT |
| 7SK_126-162D2 | TCGGGGAAGGTCGTCCTCTGACCGAGCGCGCAGCT |
| 7SK_126-162MM1 | TCGGGGAAGGTCGTCCTC**A**TCGACCGAGCGCGCAGCT |
| 7SK_126-162MM2 | TCGGGGAAGGT**G**GTCCTC**A**TCGACCGAGCGCGCAGCT |
| 7SK_126-162_RC | AGCTGCGCGCTCGGTCGAAGAGGACGACCTTCCCCGA |
| 7SK_17-63 | AGCCGAATCAACCCTGGCGATCAATGGGGTGACAGATGTCGCAGCCA |
| 7SK_17-63MM1 | AGCCGAATCAACCCTGGCGATCA**T**TGGGGTGACAGATGTCGCAGCCA |
| 7SK_17-63MM2 | AGCCGAATCAACCCTG**C**CGATCA**T**TGGGGTGACAGATGTCGCAGCCA |
| 7SK_17-63_RC | TGGCTGCGACATCTGTCACCCCATTGATCGCCAGGGTTGATTCGGCT |
| 7SK_55-91 | AGGGGACACCCGCCTAGCCAGCCAGATCAGCCGAATC |
| 7SK_55-91MM1 | AGGGGACACCCGCCTAGC**G**AGCCAGATCAGCCGAATC |
| 7SK_55-91MM2 | AGGGGACACC**G**GCCTAGC**G**AGCCAGATCAGCCGAATC |
| 7SK_55-91_RC | GATTCGGCTGATCTGGCTGGCTAGGCGGGTGTCCCCT |
| SNORA18_4-47 | CAACGACGTCTGGCTACTAGGGAAGCCCCGTCGGGATAGACCTC |
| SNORA18_4-47MM1 | CAACGACGTCTGGCTACTAGG**C**AAGCCCCGTCGGGATAGACCTC |
| SNORA18_4-47MM2 | CAACGACGTCTGGCTACTAGG**C**AAGCCCCGTCG**C**GATAGACCTC |
| SNORA18_4-47_RC | GAGGTCTATCCCGACGGGGCTTCCCTAGTAGCCAGACGTCGTTG |
| SNORA18_66-112 | CCCACAGACCTAACAGTCAGTCCTGTGAGTCAAGTAAAACCACAAGT |
| SNORA18_66-112MM1 | CCCACAGACCTAACAGTCAGTCC**A**GTGAGTCAAGTAAAACCACAAGT |
| SNORA18_66-112MM2 | CCCACAGACCTAACAGTCAGTCC**A**GTGAGTCAAGTAAAAC**G**ACAAGT |
| SNORA18_66-112_RC | ACTTGTGGTTTTACTTGACTCACAGGACTGACTGTTAGGTCTGTGGG |
| SNORA71_14-66 | GCATCCTCTCCCTGCAGTATCAATGACCAGGGCACCGGCAGCCCACAATCACT |
| SNORA71_14-66MM1 | GCATCCTCTCCCTGCAGTATCAATGA**G**CAGGGCACCGGCAGCCCACAATCACT |
| SNORA71_14-66MM2 | GCATCCTCTC**G**CTGCAGTATCAATGA**G**CAGGGCACCGGCAGCCCACAATCACT |
| SNORA71_14-66_RC | AGTGATTGTGGGCTGCCGGTGCCCTGGTCATTGATACTGCAGGGAGAGGATGC |
| SNORA71_71-124 | AAGCACCAGGGTTTGGCAAGGACAAGTGTGATCCTCCAAACACGGGGAAAGGCT |
| SNORA71_71-124MM1 | AAGCACCAGGGTTTGGCAAGGACAAG**A**GTGATCCTCCAAACACGGGGAAAGGCT |
| SNORA71_71-124MM2 | AAGCACCAG**C**GTTTGGCAAGGACAAG**A**GTGATCCTCCAAACACGGGGAAAGGCT |
| SNORA71_71-124_RC | AGCCTTTCCCCGTGTTTGGAGGATCACACTTGTCCTTGCCAAACCCTGGTGCTT |
| SNORD113_19-72 | GTGAACCTCAGACTCTCAGACGCATAGTCATAATACACGACTCATACAGCACCC |
| SNORD113_19-72MM1 | GTGAACCTCAGACTCTCAGACGCATA**C**TCATAATACACGACTCATACAGCACCC |
| SNORD113_19-72MM2 | GTGAACCTCAGACTCTCAGACGCATA**C**TCATAATACACGACT**G**ATACAGCACCC |
| SNORD113_19-72_RC | GGGTGCTGTATGAGTCGTGTATTATGACTATGCGTCTGAGAGTCTGAGGTTCAC |
| SNORD113_9-67 | CCTCAGACTCTCAGACGCATAGTCATAATACACGACTCATACAGCACCCACAGTCATCA |
| SNORD113_9-67MM1 | CCTCAGACTCTCAGACGCATAGTCATAAT**T**CACGACTCATACAGCACCCACAGTCATCA |
| SNORD113_9-67MM2 | CCTCAGACTCTCAGAC**C**CATAGTCATAAT**T**CACGACTCATACAGCACCCACAGTCATCA |
| SNORD113_9-67_RC | TGATGACTGTGGGTGCTGTATGAGTCGTGTATTATGACTATGCGTCTGAGAGTCTGAGG |
| SNORD55_29-75 | CGCTCAGCTCTCCAAGGTTGGCTTCCCCACGACGCAGTCGGGAGTGT |
| SNORD55_29-75MM1 | CGCTCAGCTCTCCAAGGTTGGCT**A**CCCCACGACGCAGTCGGGAGTGT |
| SNORD55_29-75MM2 | CGCTCAGCTCTCCAAGGTTGGCT**A**CCCCACGACG**G**AGTCGGGAGTGT |
| SNORD55_29-75_RC | ACACTCCCGACTGCGTCGTGGGGAAGCCAACCTTGGAGAGCTGAGCG |
| SNORD55_3-41 | CAGTCGGGAGTGTGCAGCATACCCAGGTGTCATCATCCG |
| SNORD55_3-41MM1 | CAGTCGGGAGTGTGCAGCA**A**ACCCAGGTGTCATCATCCG |
| SNORD55_3-41MM2 | CAGTCGGGA**C**TGTGCAGCA**A**ACCCAGGTGTCATCATCCG |
| SNORD55_3-41_RC | CGGATGATGACACCTGGGTATGCTGCACACTCCCGACTG |
| SNORD81_10-58 | CTGTCATCAAGTAATCAGTGAGAGAGTCCAAGTTGGGGTGAGATCATCA |
| SNORD81_10-58MM1 | CTGTCATCAAGTAATCAGTGAGAG**T**GTCCAAGTTGGGGTGAGATCATCA |
| SNORD81_10-58MM2 | CTGTCATCAAGTAATCAGTGAGAG**T**GTCCAAGTTGGGGTGA**C**ATCATCA |
| SNORD81_10-58_RC | TGATGATCTCACCCCAACTTGGACTCTCTCACTGATTACTTGATGACAG |
| SNORD81_2-58 | CTGTCATCAAGTAATCAGTGAGAGAGTCCAAGTTGGGGTGAGATCATCATGTGTCCT |
| SNORD81_2-58MM1 | CTGTCATCAAGTAATCAGTGAGAGAGTC**G**AAGTTGGGGTGAGATCATCATGTGTCCT |
| SNORD81_2-58MM2 | CTGTCATCAAGTAATCA**C**TGAGAGAGTC**G**AAGTTGGGGTGAGATCATCATGTGTCCT |
| SNORD81_2-58_RC | AGGACACATGATGATCTCACCCCAACTTGGACTCTCTCACTGATTACTTGATGACAG |
| SNOZ39_6-60 | TACATCAGAAAGCGTTTACAGTATCATTTTATTCATACTGATTTGCTTCATCACA |
| SNOZ39_6-60MM1 | TACATCAGAAAGCGTTTACAGTATCAT**A**TTATTCATACTGATTTGCTTCATCACA |
| SNOZ39_6-60MM2 | TACATCAGAAAGCGTTTACAGTATCAT**A**TTATTCATACTGATTT**C**CTTCATCACA |
| SNOZ39_6-60_RC | TGTGATGAAGCAAATCAGTATGAATAAAATGATACTGTAAACGCTTTCTGATGTA |
| tRNALys_1-59 | CTCGAACCCACGACCCTGGGATTAAGAGTCCCATGCTCTACCGACTGAGCTAGCCGGGC |
| tRNALys_1-59D1 | CTCGAACCCACGACCCTGGGATTAAGAGCCCATGCTCTACCGACTGAGCTAGCCGGGC |
| tRNALys_1-59D2 | CTCGAACCCACGACCCTGGGATTAAGAGCCATGCTCTACCGACTGAGCTAGCCGGGC |
| tRNALys_1-59MM1 | CTCGAACCCACGACCCTGGGATTAAGAGT**G**CCATGCTCTACCGACTGAGCTAGCCGGGC |
| tRNALys_1-59MM2 | CTCGAACCCACGACCCTGGGATTAAGAGT**G**CCATGCTCTACC**C**ACTGAGCTAGCCGGGC |
| tRNALys_1-59_RC | GCCCGGCTAGCTCAGTCGGTAGAGCATGGGACTCTTAATCCCAGGGTCGTGGGTTCGAG |
| tRNALys_40-69 | CAACGTGGGGCTCGAACCCACGACCCTGGG |
| tRNALys_40-69MM1 | CAACGTGGGGCTCG**T**ACCCACGACCCTGGG |
| tRNALys_40-69MM2 | CAACGT**C**GGGCTCG**T**ACCCACGACCCTGGG |
| tRNALys_40-69_RC | CCCAGGGTCGTGGGTTCGAGCCCCACGTTG |
| tRNAPhe_16-75 | GGTGCCGAAACCCGGGATCGAACCAGGGACCTTTAGATCTTCAGTCTAACGCTCTCCCAA |
| tRNAPhe_16-75MM1 | GGTGCCGAAACCCGGGATCGAACCAGGGA**G**CTTTAGATCTTCAGTCTAACGCTCTCCCAA |
| tRNAPhe_16-75MM2 | GGTGCCGAAACCCGGGATCGAACCAGGGA**G**CTTTAGATCTTCA**C**TCTAACGCTCTCCCAA |
| tRNAPhe_16-75_RC | TTGGGAGAGCGTTAGACTGAAGATCTAAAGGTCCCTGGTTCGATCCCGGGTTTCGGCACC |
| tRNAPhe_47-76 | TGGTGCCGAAACCCGGGATCGAACCAGGGA |
| tRNAPhe_47-76MM1 | TGGTGCCGAAACCC**C**GGATCGAACCAGGGA |
| tRNAPhe_47-76MM2 | TGGT**C**CCGAAACCC**C**GGATCGAACCAGGGA |
| tRNAPhe_47-76_RC | TCCCTGGTTCGATCCCGGGTTTCGGCACCA |

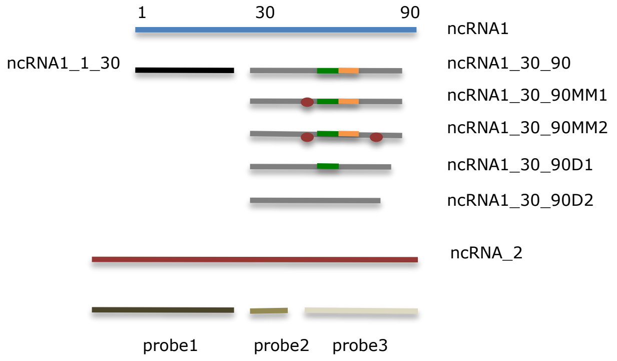


Supplementary Table 1

| UniqueID | Name | ES vs Brain total RNA |
|---|---|---|
| 1L17 | hsa_SNORD13 | -2.0010154 |
| 1N18 | hsa_SNORD2 | -1.9591408 |
| 1M18 | hsa_SNORD3@ | -1.3929354 |
| 5N14 | hsa-let-7a | 3.9104207 |
| 5C8 | hsa-miR-106a | -2.200453 |
| 2L11 | hsa-miR-371-5p | -2.7227075 |
| 1D17 | hsa-miR-498 | -1.9325277 |
| 1I17 | hsa-miR-503 | -1.1462102 |
| 2A5 | hsa-miR-659 | -1.0401192 |
| 2H5 | hsa-miR-9* | 3.751164 |
| 2G14 | miRPlus_17890 | -1.3811024 |
| 2I13 | miRPlus_17952 | -1.176801 |
| 5D2 | miRPlus_21472 | -1.5241518 |
| 5E4 | miRPlus_27561 | -2.174772 |
| 3E18 | mmu-let-7a | 3.8383002 |
| 5H14 | mmu-let-7b | 1.044384 |
| 1C1 | mmu-let-7c | 2.6271546 |
| 5D14 | mmu-let-7d | 3.1397784 |
| 1E1 | mmu-let-7e | 1.5860898 |
| 1G1 | mmu-let-7g | 3.562132 |
| 1H1 | mmu-let-7i | 4.08837 |
| 1L1 | mmu-miR-103 | 2.9407806 |
| 5G7 | mmu-miR-107 | 3.2431517 |
| 1G2 | mmu-miR-124 | 3.7746544 |
| 1H2 | mmu-miR-125a-5p | 2.6573153 |
| 5N11 | mmu-miR-125b-5p | 3.75356 |
| 5A14 | mmu-miR-128a/128b | 3.7520082 |
| 2E7 | mmu-miR-129-3p | 2.9990404 |
| 1I3 | mmu-miR-138 | 3.6429393 |
| 1A5 | mmu-miR-16 | 1.7949975 |
| 5B8 | mmu-miR-17 | -2.3353066 |
| 1E6 | mmu-miR-191 | 1.9610482 |
| 1L6 | mmu-miR-195 | 3.7988846 |
| 1J7 | mmu-miR-200b | 1.7533876 |
| 1F7 | mmu-miR-20a | -2.3012671 |
| 1L8 | mmu-miR-218 | 3.9354165 |
| 1N8 | mmu-miR-22 | 2.191882 |

| UniqueID | Name | ES vs Brain total RNA |
|---|---|---|
| 5B7 | mmu-miR-23a | 2.4401088 |
| 1E9 | mmu-miR-23b | 2.5244708 |
| 1F9 | mmu-miR-24 | 2.584994 |
| 1H9 | mmu-miR-26a | 3.1850955 |
| 1K9 | mmu-miR-27b | 2.5625648 |
| 6M5 | mmu-miR-291b-5p | -3.5358765 |
| 2I18 | mmu-miR-292-3p | -4.7475047 |
| 2K18 | mmu-miR-293 | -4.643447 |
| 2L18 | mmu-miR-294 | -4.5158563 |
| 2M18 | mmu-miR-295 | -4.7702394 |
| 1P9 | mmu-miR-29a | 3.3800652 |
| 1L10 | mmu-miR-30a | 3.1322336 |
| 1M10 | mmu-miR-30b | 3.0904121 |
| 1N10 | mmu-miR-30c | 2.9647126 |
| 1O10 | mmu-miR-30d | 3.6222904 |
| 1O11 | mmu-miR-335-5p | 2.2869596 |
| 1A12 | mmu-miR-338-3p | 2.3757057 |
| 5N9 | mmu-miR-369-3p | 2.4375849 |
| 5J10 | mmu-miR-374 | 1.8789672 |
| 2C21 | mmu-miR-467b | -1.3100047 |
| 5H2 | mmu-miR-668 | -1.0478611 |
| 2H22 | mmu-miR-677 | -2.3486063 |
| 2I22 | mmu-miR-678 | -1.3342453 |
| 2P22 | mmu-miR-685 | -1.1779056 |
| 2D23 | mmu-miR-689 | -3.6705205 |
| 2E24 | mmu-miR-706 | -2.4139588 |
| 5D9 | mmu-miR-708 | 1.1890153 |
| 2G5 | mmu-miR-9 | 3.8342102 |
| 5E14 | mmu-miR-9* | 2.2085142 |
| 2N5 | mmu-miR-98 | 3.8455708 |
| 7J2 | SNORA71_14-66 | -2.1912198 |
| 7K2 | SNORA71_14-66MM1 | -2.1310618 |
| 7N2 | SNORA71_71-124 | -2.476882 |
| 7O2 | SNORA71_71-124MM1 | -2.3021042 |
| 7P2 | SNORA71_71-124MM2 | -2.4302258 |
| 7N3 | SNORD55_3-41 | -1.0061008 |
| 1A2 | U6-snRNA-1 | -1.3984196 |
| 1B2 | U6-snRNA-2 | -1.2016013 |

# Supplementary Table 2
